# Retraction: Biosurfactant producing multifarious *Streptomyces puniceus* RHPR9 of *Coscinium fenestratum* rhizosphere promotes plant growth in chilli

**DOI:** 10.1371/journal.pone.0273533

**Published:** 2022-08-31

**Authors:** The PLOS ONE Editors

The *PLOS ONE* Editors retract this article [1] because it was identified as one of a series of submissions for which we have concerns about authorship, competing interests, and peer review. We regret that the issues were not addressed prior to the article’s publication.

DR, NAB, and MYK either did not respond directly or could not be reached. PR, MM, AAH, RMS, BH, HAEE, SZH, and RZS did not agree with the retraction.
